# Determinants of oncologic outcomes in high‐grade organ‐confined prostate cancer after prostatectomy

**DOI:** 10.1111/his.15547

**Published:** 2025-08-29

**Authors:** Reem Youssef, Omer AM Saeed, Ezra Baraban, Mohammad Salimian, Kenneth A Iczkowski, Lorene J Chung, Nicholas Baniak, Eva M Compérat, Ying Wang, Geert JLH van Leenders, Ankur R Sangoi, Douglas Jian‐Xian Wu, Adeboye O Osunkoya, Shivani Kandukuri, Alicia Cuber, Kvetoslava Michalova, Andrea Strakova‐Peterikova, Guido Martignoni, Anna Caliò, Lisa Marcolini, Hiroshi Miyamoto, Angela Pecoraro, Toyonori Tsuzuki, Andres Martin Acosta

**Affiliations:** ^1^ Department of Pathology Indiana University School of Medicine Indianapolis Indiana USA; ^2^ Department of Pathology Johns Hopkins Medical Institutions Baltimore Maryland USA; ^3^ Department of Pathology University of California—Davis Sacramento California USA; ^4^ Department of Pathology University of Saskatchewan Saskatoon Saskatchewan Canada; ^5^ Department of Pathology Medical University of Vienna Vienna Austria; ^6^ Department of Pathology & Laboratory Medicine University of Rochester Medical Center Rochester New York USA; ^7^ Department of Pathology Erasmus MC Cancer Institute Rotterdam The Netherlands; ^8^ Department of Pathology Stanford University Stanford California USA; ^9^ Department of Pathology Emory University School of Medicine Atlanta Georgia USA; ^10^ Department of Pathology Keck School of Medicine of USC Los Angeles California USA; ^11^ Department of Pathology Charles University, Faculty of Medicine in Plzeň, Bioptical Laboratory, Ltd. Pilsen Czech Republic; ^12^ Department of Diagnostics and Public Health, Section of Pathology University of Verona Verona Italy; ^13^ Department of Pathology Pederzoli Hospital Verona Italy; ^14^ Department of Urology Pederzoli Hospital Verona Italy; ^15^ Department of Pathology Aichi Medical University Hospital Nagakute Aichi Japan

**Keywords:** cribriform, Grade Group 4, Grade Group 5, high‐grade prostate cancer, high‐risk prostate cancer, organ‐confined, prostate cancer, pT2

## Abstract

**Aims:**

In radical prostatectomy (RP), Grade Group (GG) 4/5 prostate cancer [high‐grade prostate cancer (HGPC) hereafter] is often associated with extension beyond the prostate and positive surgical margins. Hence, there is limited information on post‐RP outcomes of patients with completely resected HGPC confined to the prostate (pT2).

**Materials and methods:**

Clinical outcomes were assessed in a cohort of patients with pT2 HGPC and negative surgical margins using Kaplan‐Meier statistics and Cox regression analysis.

**Results and conclusion:**

Four hundred and seven RPs were initially assessed: 236 (58%) with GG 4 and 171 (42%) with GG 5 prostate cancer (PCa). Survival analysis was performed on subsets of patients with available follow‐up (BCR: *n* = 343, metastases: *n* = 347) to identify clinicopathologic variables associated with the risk of biochemical recurrence and metastasis. The size of the dominant nodule (cut‐off 15 mm) (HR 1.654, 95% CI 1.026–2.667; *P* = 0.04) and the preoperative PSA level (HR 1.052, 95% CI 1.009–1.097; *P* = 0.02) were associated with a higher likelihood of BCR on univariate regression analysis, with only preoperative PSA remaining significant when both variables were assessed concurrently (HR 1.051, 95% CI 1.007–1.098; *P* = 0.02). On univariate Cox regression analysis, the size of the dominant nodule (cut‐off: 15 mm; HR 6.315, 95% CI 2.021–19.725; *P <* 0.01), the presence of large cribriform components (HR 4.375, 95% CI 0.999–19.159; *P* = 0.05), and LVI (HR 3.808, 95% CI 1.086–13.354; *P* = 0.04) were associated with the risk of metastasis, but only size remained an independent predictor on multivariate analysis (HR 5.66, 95% CI 1.761–18.191; *P* < 0.01In p for cut‐off of 15 mm).

## Graphical abstract

In patients with pT2 HGPC and negative margins, both the size of the dominant nodule (cut‐off = 15 mm) and the preoperative PSA levels are associated with a higher risk of BCR, with preoperative PSA levels representing an independent predictor. Additionally, cribriform PCa, LVI, and a tumour size seem to portend a higher risk of metastasis, with size remaining an independent predictor when these variables are assessed concurrently.

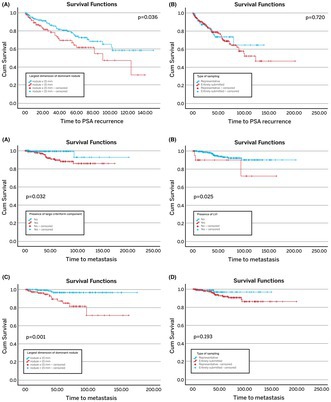

**Abbreviations**
BCR: biochemical recurrence
GG: grade group
GP: Gleason pattern
HGPC: high grade prostate cancer
LVI: lymphovascular iinvasion
PCa: prostate cancer
PNI: perineural invasion
PSA: prostate specific antigen
RP: radical prostatectomy

## Introduction

Histologic grading, one of the strongest predictors of clinical outcomes in prostate cancer (PCa), is a challenging yet essential process for guiding treatment. Studies have shown that Grade Group (GG) 4–5 prostate cancer [high‐grade prostate cancer (HGPC) hereafter] is associated with unfavourable disease‐specific outcomes,^1^ showing a high frequency of extraprostatic extension, seminal vesicle invasion, and surgical margin involvement, especially when it contains cribriform or solid components.^2^, ^3^ However, HGPC is not a uniform entity, as it comprises a heterogeneous group of lesions with various combinations of growth patterns.

Over the last decade, multiple studies have shown that, within Gleason pattern (GP) 4, cribriform morphology is associated with a high risk of biochemical recurrence (BCR) and metastasis after radical prostatectomy (RP).^2^, ^4^ Similarly, within GP5, the presence of large solid growth pattern and comedonecrosis has been associated with poor prognosis and a higher likelihood of BCR and metastasis post‐RP.^5^ The risk of BCR in HGPC after RP is also strongly influenced by the pathologic stage, with tumours that extend beyond the prostate (pT3a and pT3b) showing higher rates of PSA recurrence than those that are organ confined (pT2).^2^ Given that most HGPC present with an advanced local stage^6^ and, historically, non‐surgical options such as combination radiotherapy and androgen deprivation were favoured over RP for patients with high‐risk prostate cancer,^7^ information on clinical outcomes of patients with completely resected organ confined (pT2) HGPC is limited. In this study, we evaluated the risk of BCR and metastases after RP in patients with completely resected HGPC confined to the prostate.

## Materials and Methods

This research was performed with the approval of the Institutional Review Board of Indiana University (protocol #18697, 2023).

### Accrual of Cases and Collection of Clinical and Pathology Data

RPs with HGPC confined to the prostate (pT2 pN0 cM0) and no history of neoadjuvant treatment or prior radiation (including brachytherapy) were gathered retrospectively from the participating institutions. Slides of the RPs with pT2 HGPC were reviewed on‐site (i.e. at the participating institutions) by genitourinary pathologists to assess grade, stage and margin status. To mitigate the lack of a central review, a document with definitions of inclusion criteria, variables of interest and outcomes was provided to all participants (see supplemental material). In addition to grade and stage, the following histopathologic variables were gathered: largest dimension of the dominant nodule (the nodule with the highest Gleason score/Grade Group), location of the dominant nodule, configuration of the dominant nodule (well‐circumscribed vs. diffuse), sampling mode (representatively vs. entirely submitted), large cribriform architecture (defined as cribriform glands ≥0.25 mm in diameter or measuring at least twice the size of adjacent benign glands),^8^, ^9^ lymphovascular invasion (LVI), and perineural invasion (PNI). Both intraductal and invasive cribriform glands were considered to represent large cribriform components if they met size criteria. Size was obtained from the largest dimension annotated in the original pathology report or, alternatively, as the largest linear dimension measured on glass slides. Well‐circumscribed tumour nodules were defined by the presence of an expansile appearance and sharp borders. The following clinical and demographic data were collected: age at RP, preoperative PSA, adjuvant treatment received (hormonal, radiotherapy or combined) and clinical outcomes (BCR, development of metastases, and disease‐specific death). Time to clinical outcome was computed from the time of RP; patients without the event of interest were censored at the time of last follow‐up for survival analysis (see below). Metastases were defined as biopsy‐proven lesions and/or clinical/imaging findings considered diagnostic of metastatic disease by the treating clinician. Death of disease was defined as death as a direct result of cancer progression.

### Statistical Analyses

Distribution of values for demographic and clinicopathologic variables was presented as median and range for continuous variables and as frequencies (percentages) for categorical variables. Probability of clinical outcomes was assessed with Kaplan–Meier curves and Log Rank test. Cases were censored at the time of last follow‐up if the outcomes of interest were not observed. Additionally, univariable and multivariable logistic Cox regression models were used to assess the association between variables and clinical outcomes. Covariates included Gleason Grade Group, preoperative PSA, largest dimension of the dominant nodule, configuration of the dominant nodule, sampling mode, cribriform status, LVI and PNI. Clinical outcomes included BCR and development of metastasis (there were too few events to assess disease‐specific mortality; see results below). *P* < 0.05 was considered the threshold for statistical significance. Analyses of clinical outcomes and survival were computed with SPSS software (version IBM SPSS Statistics 29).

## Results

### Demographic and Clinical Characteristics

**Figure 1:**
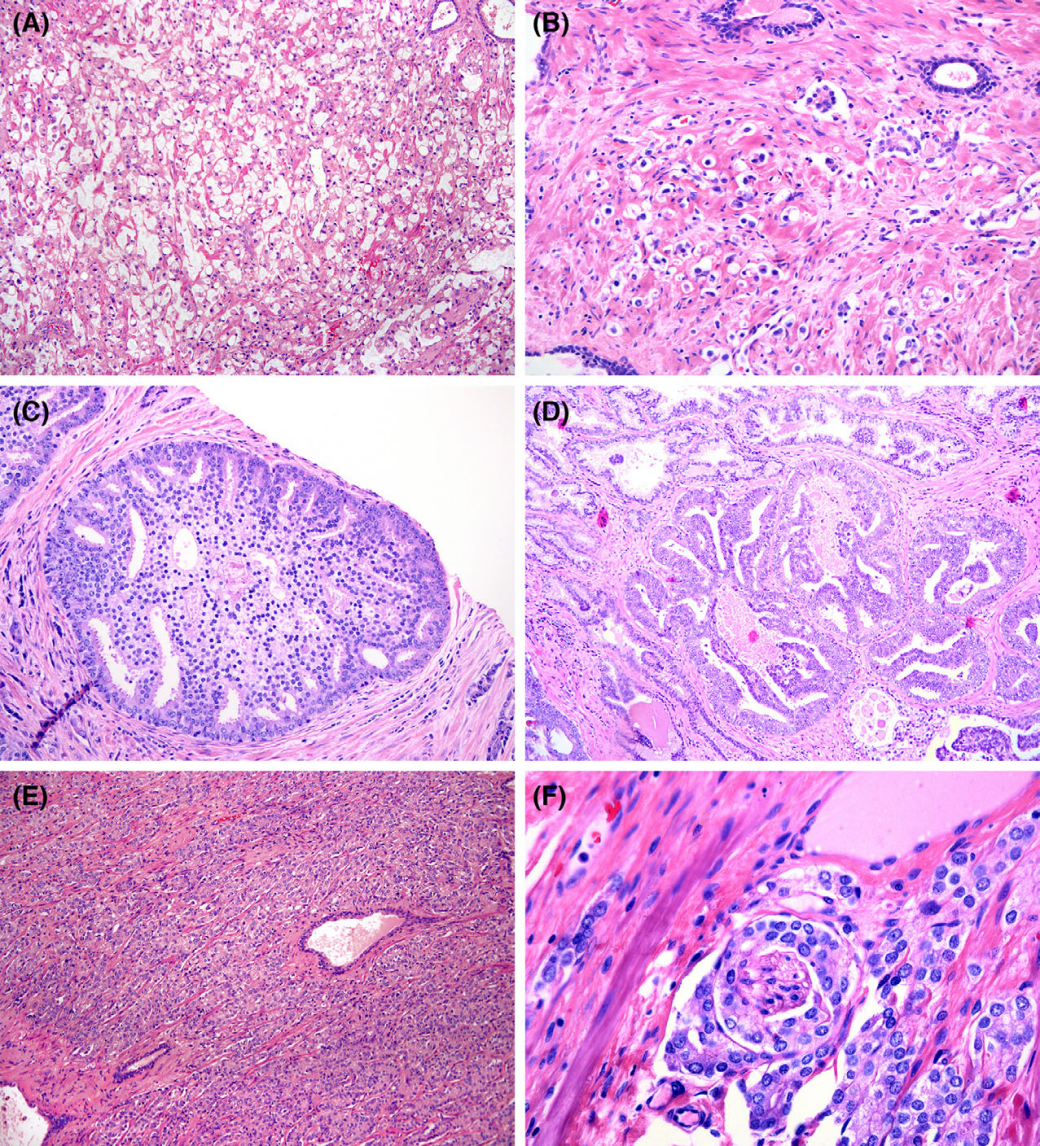
**(A–F)** Histologic features of high‐grade prostate cancer (GG4/GG5). **(A)** Gleason pattern 5 with vacuolated tumor cells (10×). **(B)** Gleason pattern 5 with single‐cell growth pattern (20×). **(C)** Gleason pattern 4 with large cribriform cancer glands (20×). **(D)** Cribriform carcinoma with comedonecrosis (10×). **(E)** Gleason pattern 4 with poorly formed and fused glands (10X). **(F)** High‐magnification view of a focus of perineural invasion, one of the variables included in the analyses (40×).

The series comprised 407 patients who underwent RP and fulfilled the inclusion criteria defined above (representative tumours included in the series are shown in Figure 1). The median age was 66 years (range: 41–86 years), and the median preoperative PSA was 7.20 ng/ml (range: 0.74–76). Only 13 (3.4%) patients in the series showed preoperative PSA levels higher than 20 ng/mL, including 9 with PSA between 20 and 30 ng/mL, 3 with PSA between 30 and 40 ng/mL and 1 with PSA of 76 ng/mL.

### Pathologic Characteristics

Among the 407 RP, 236 (58%) showed GG4 and 171 (42%) showed GG5 PCa. In 324 patients (80%), the prostate was entirely submitted, while the remaining 83 prostates (20%) were representatively sampled. The median dimension of the largest dominant nodule was 15 mm (range: 1–50 mm). The location of the dominant nodule was difficult to determine precisely in multiple cases and was therefore excluded from the survival analysis. Based on the available annotations, 48 nodules (11.8%) appear to be exclusively or predominantly anterior.

### Clinical Outcomes

Survival analysis was performed only in cases with available follow‐up (BCR: *n* = 343, metastases: *n* = 347).

**Figure 2:**
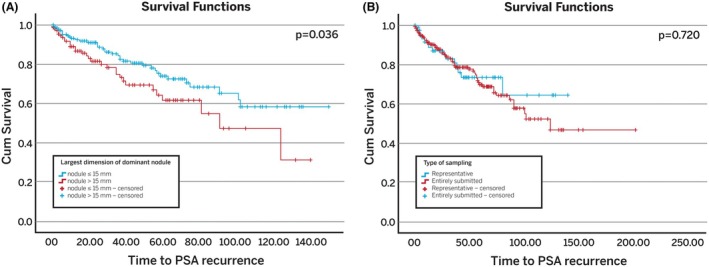
Kaplan–Meier statistics: Biochemical recurrence (BCR) in radical prostatectomies with pT2 high‐grade prostate cancer (GG4/5). **(A)** BCR probability stratified according to the size of largest dimension of the dominant nodule (15 mm and below vs. more than 15 mm). **(B)** BCR probability stratified according to the type of sampling (representatively vs entirely submitted).

Kaplan–Meier analysis showed that a tumour size >15 mm (median size of the dominant nodule) was associated with a higher risk of PSA recurrence (*P* = 0.036; Figure 2A). No differences in risk of BCR or metastases were observed between patients in whom RPs were representative and entirely submitted (Figure 2B). Univariate Cox regression analysis showed that both a dominant nodule size >15 mm (HR 1.654, CI 95% 1.026–2.667; *P* = 0.04) and the preoperative PSA level (HR 1.052, 95% CI 1.009–1.097; *P* = 0.02) were associated with a higher likelihood of BCR. However, only the preoperative PSA level remained significant when both variables were considered concurrently (HR 1.051, 95% CI 1.007–1.098; *P* = 0.02) (Table 1).

**Table 1:** Cox proportional hazard models of biochemical recurrence

	Univariate analysis Cox regression	Multivariate analysis Cox regression		
	HR (95% CI)	*P*	HR (95% CI)	*P*
Sampling	1.107 (0.635–1.929)	0.72	‐	‐
Configuration of the dominant nodule	1.119 (0.690–1.815)	0.65	‐	‐
WHO/ISUP grade	1.074 (0.678–1.703)	0.76	‐	‐
Large cribriform components	1.581 (0.944–2.648)	0.08	‐	‐
LVI	1.203 (0.484–2.99)	0.69	‐	‐
Perineural invasion	0.934 (0.577–1.513)	0.78	‐	‐
Largest dimension of the dominant nodule	1.019 (0.987–1.053)	0.24	‐	‐
Largest dimension of nodule (15 and below vs. more than 15)	1.654 (1.026–2.667)	0.04	1.449 (0.890–2.361)	0.14
Preoperative PSA level	1.052 (1.009–1.097)	0.02	1.051 (1.007–1.098)	0.02

CI, confidence interval; HR, hazard ratio; ISUP, International Society of Urological Pathology; LVI, lymphovascular invasion; PSA, prostate specific antigen; WHO, World Health Organization.

**Figure 3:**
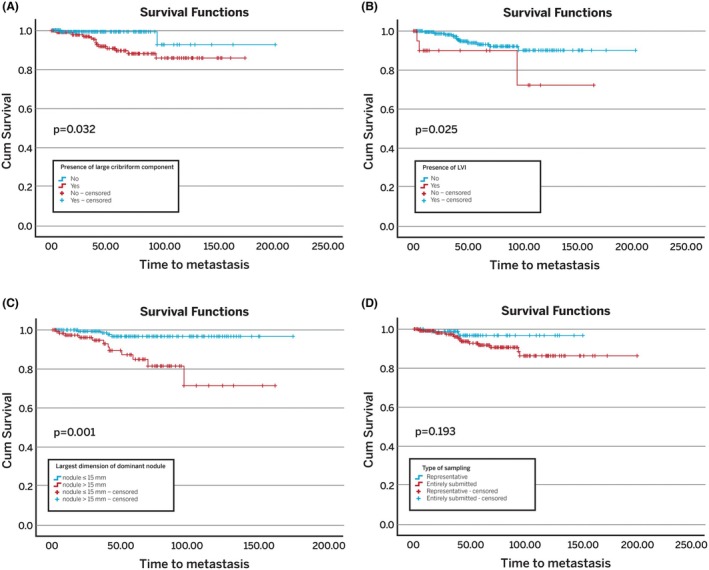
Kaplan–Meier statistics: Metastasis in radical prostatectomies with pT2 high‐grade prostate cancer (GG4/5). **(A)** Metastasis probability stratified according to the presence or absence of large cribriform components. **(B)** Metastasis probability stratified according to the presence or absence of lymphovascular invasion. **(C)** Metastasis probability stratified according to the size of the largest dimension of the dominant nodule (15 mm and below vs. more than 15 mm). **(D)** Metastasis probability stratified according to the type of sampling (representatively vs. entirely submitted).

Kaplan–Meier analysis demonstrated that the presence of large cribriform components (*P* = 0.032), LVI (*P* = 0.025), and a tumour size >15 mm (*P* = 0,001) were associated with a higher risk of developing metastases (Figure 3). No differences in the risk of metastases were observed between patients in whom RPs were representative and entirely submitted (Figure 3D). Univariate Cox regression analysis showed that large cribriform components (HR 4.375, 95% CI 0.999–19.159; *P* = 0.05), LVI (HR 3.808, 95% CI 1.086–13.354; *P* = 0.04) and size of the dominant nodule (HR 1.117, 95% CI 1.060–1.177; *P* < 0.01) were all associated with a higher likelihood of developing metastasis. Only the size of the dominant nodule remained significant when these variables were assessed concurrently (multivariate Cox regression analysis: HR 1.108, 95% CI 1.045–1.174; *P* < 0.01 for continuous size; HR 5.66, 95% CI 1.761–18.191; *P* < 0.01 for size 0–15 mm vs. >15 mm) (Table 2). Too few events were present to evaluate disease‐specific mortality; therefore, this outcome was excluded from the analysis.

**Table 2:** Cox proportional hazard models of development of metastasis

	Univariate analysis Cox regression	Multivariate analysis Cox regression		
	HR (95% CI)	*P*	HR (95% CI)	*P*
Sampling	2.572 (0.587–11.273)	0.21	_	_
Configuration of the dominant nodule	2.499 (0.888–7.033)	0.08	_	_
WHO/ISUP grade	2.420 (0.893–6.558)	0.08	_	_
Large cribriform components	4.375 (0.999–19.159)	0.05	3.392 (0.766–15.032)	0.11
LVI	3.808 (1.086–13.354)	0.04	1.271 (0.248–6.510)	0.77
Perineural invasion	2.931 (0.840–10.221)	0.09	_	_
Largest dimension of the dominant nodule (Scale)	1.117 (1.060–1.177)	< 0.01	1.108 (1.045–1.174)	< 0.01
Largest dimension of nodule (15 and below vs. more than 15)	6.315 (2.021–19.725)	< 0.01	5.66 (1.761–18.191)	< 0.01
Preoperative PSA level	1.054 (0.972–1.143)	0.25	_	_

CI, confidence interval; HR, hazard ratio; ISUP, International Society of Urological Pathology; LVI, lymphovascular invasion; PSA, prostate specific antigen; WHO, World Health Organization.

## Discussion

The Gleason score and resulting Grade Groups remain among the strongest predictors of prognosis in patients with PCa.^1^ HGPC (Gleason scores 8–10; GG 4–5) is associated with unfavourable outcomes, with a high frequency of BCR after treatment performed with curative intent. More specifically, patients with GG4 PCa show a 5‐year BCR‐free survival (BCR‐FS) rate of ~64%; whereas BCR‐free survival is only 35% in those with GG5 PCa.^1^ These high‐grade tumours tend to show advanced local pathologic stage (i.e., extension beyond the prostate) and involvement of surgical resection margins, findings that contribute to their poor clinical outcomes.^2^, ^5^ In GG >2 PCa, extension beyond the prostate is an independent predictor of BCR and disease progression/progression‐free survival.^10^, ^11^ The presence of transected tumour at the surgical margin is also an independent predictor of BCR, with risk being positively correlated with the extent of margin involvement.^12^ Given that most (>70%) HGPCs present at an advanced local stage^13^ and show a high frequency of margin involvement, there is limited knowledge about the clinical behaviour of HGPC that is confined to the prostate and completely resected. Many prior studies of RPs that comprised organ‐confined GG4‐5 PCa included cases with lymph node invasion and positive surgical margins.^14^, ^15^ A study by Preisser *et al*. evaluated the outcomes of organ‐confined GG4‐5 PCa with negative surgical margins following RP. In this study, GG4‐5 PCa represented only 1.8% of all pT2 RPs (195 of 10,855 patients).^16^ Ten‐year BCR‐free survival of GG4 and GG5 PCa at 10 was ~69% and ~55%, respectively; whereas 10‐year metastasis‐free survival was ~90% (GG4) and ~83% (GG5), and 10‐year cancer‐specific survival was ~98% (GG4) and 83% (GG5).^16^ This study also found that GG5 was an independent predictor of BCR and metastases and that both GG4 and GG5 were independently associated with cancer‐specific mortality.^16^ Rioux‐Leclercq *et al*. studied disease‐specific outcomes in 27 patients with RP showing HGPC confined to the prostate, documenting a rate of disease progression of 32% (10/27).^17^ Interestingly, the authors found that progression was not associated with any of the clinicopathologic variables that were assessed. In recent years, cribriform PCa, including both invasive and intraductal carcinoma with cribriform architecture, has been identified as a major predictor of clinical outcomes in PCa across Grade Groups.^2^, ^4^, ^18^, ^19^ Specifically, cribriform PCa is associated with multiple adverse histopathologic findings on RP and represents an independent predictor of BCR and metastasis.^2^, ^4^, ^19^, ^20^, ^21^ Three‐dimensional RP tissue reconstructions performed by Verhoef *et al*. have revealed that there is a spatial continuum between cribriform and solid components of PCa, suggesting that the latter may show similar clinical implications.^22^ Accordingly, recent analyses of RP with HGPC have shown that the presence of areas with solid growth and comedonecrosis (which is commonly seen in solid components) is associated with multiple adverse histopathologic findings as well as with higher risk of BCR and metastasis.^2^, ^5^, ^23^

Prior studies of organ‐confined HGPC assessed a limited number of histopathologic variables and did not include cribriform components in the analyses.^16^, ^17^ This large study of organ‐confined HGPC found that, in RPs with confirmed pT2 stage and negative surgical margins, BCR and metastases occurred in 21.2% and 4.8% of patients, respectively. However, these figures almost certainly underestimate the true frequency of BCR and metastasis due to the limited follow‐up of the cohort (median = 42 months). A dominant nodule size >15 mm and the preoperative PSA level were associated with a higher risk of BCR, with the preoperative PSA level representing the only independent predictor on multivariate analysis. Cribriform components, LVI, and the size of the dominant PCa nodule were all associated with a higher risk of developing metastases; however, only the size of the nodule remained an independent predictor in multivariate analysis. This suggests that the tumour burden may be an important determinant of clinical outcomes in completely resected, organ‐confined HGPC. The lesional size typically reflects the proliferative potential and the cellular mass of the tumour. Hence, we hypothesize that, in larger tumours, a high proliferative rate and/or a large population of neoplastic cells increases the chance of emergence of subclones with metastatic potential. Although interesting, these results should be interpreted cautiously, given the limited number of events (*n* = 16 for metastases) and the limitations of the study mentioned below.

There is a longstanding debate among pathologists about the ideal method for sampling RP specimens. Although representative sampling may theoretically result in the oversight of important prognostic findings such as areas of tumour extension into extraprostatic soft tissue, surgical margin involvement and seminal vesicle involvement, it is considered an acceptable method for handling RPs.^24^ Our findings highlight that representative sampling in completely resected, organ‐confined HGPC was not associated with increased risk of adverse clinical outcomes (Figures 2B and 3D), suggesting that no adverse prognostic findings were missed. Therefore, representative sampling with complete embedding of the posterior quadrants seems appropriate, unless specific concerns arise (e.g. PCa that appears to extensively extend into an unsampled area of the prostate).

This study has important limitations that should be briefly discussed. First, it is a retrospective series with a relatively short median follow‐up time (42 months). Accordingly, the number of deaths due to PCa is too small to assess disease‐specific mortality. Second, RPs were processed at different institutions with different embedding methods (entire versus representative). Third, the potential impact of adjuvant therapy on clinical outcomes has not been addressed in this study and warrants further exploration in future research. Fourth, a single standardized method to determine the largest linear dimension of the tumour could not be used. Finally, the number of patients who developed metastases is limited. Notwithstanding these shortcomings, this large multi‐institutional study of completely resected, organ‐confined HGPC, including almost twice as many patients as the previous largest study (195 cases), provided informative results useful for clinical management.^16^ Importantly, unlike prior series, the present one assessed the prognostic value of large cribriform components in pT2 HGPC.

In conclusion, this study demonstrates that both the size of the dominant nodule (cut‐off = 15 mm) and the preoperative PSA levels are associated with a higher risk of BCR, with preoperative PSA levels representing an independent predictor. Additionally, cribriform PCa, LVI, and tumour size seem to portend a higher risk of metastasis, with size remaining an independent predictor when these variables are assessed concurrently. From a practical perspective, the present study shows that representative sampling is adequate for handling RPs with pT2 HGPCa, unless pathologic examination raises specific concerns.

## Author contributions

Concept: Andres M. Acosta. Design and coordination: Andres M. Acosta and Reem Youssef. Review of cases and data collection: All authors. Manuscript draft: Reem Youssef and Andres M. Acosta. Statistics: Omer AM Saeed. Manuscript review: All authors.

## Conflict of interests

The authors do not have financial or intellectual conflicts of interest pertaining to the contents of this article.

## Supporting information


Data S1.

## Data Availability

The data that support the findings of this study are available from the corresponding author upon reasonable request.
