# Supplementary material for: Determinants of oncologic outcomes in high‐grade organ‐confined prostate cancer after prostatectomy
Source: Histopathology. 2025 Aug 29;87(5):736–44. doi: 10.1111/his.15547 (PMC12522032; doi:10.1111/his.15547)
Supplement: Supplementary file 1 — Data S1. [file HIS-87-736-s001.docx]

**Histopathologic features and outcome of high-grade prostate cancer confined to the prostate**

Please read this short guide before completing the worksheet to ensure the homogeneity of the collected data.

*Inclusion criteria:*

1- Radical prostatectomies with Grade Group 4 or Grade Group 5 prostate cancer

**AND**

2- pT2 pN0 stage or pT2 pNx only if there is evidence to support cN0 disease

**AND**

3- Negative surgical margins

**AND**

4- No evidence of metastases at presentation (cM0)

**AND**

5- Slides are available for re-review (glass or digital)

*Exclusion criteria*

1- Any of the inclusion criteria 1-3 are not met after re-review of slides

**OR**

2- Patient received neoadjuvant therapy (or pre-operative non-neoadjuvant ADT only if there are significant treatment-induced changes)

**OR**

3- Salvage prostatectomy after radiation (including brachytherapy)

*Events collected for outcome analysis:*

1- Biochemical/PSA recurrence

2- Metastasis

3- Death of disease

*Important definitions:*

Dominant nodule: Tumor nodule with the highest Gleason score/Grade Group.

Biochemical recurrence: (1) If post-RP PSA is undetectable, at least 2 consecutive PSA values ≥ 0.2 ng/mL (ideally separated by at least 2 months) (2) If post-RP levels are detectable, at least 2 consecutive measurements (separated by at least 2 months) demonstrating rising PSA.

Metastases: (1) Biopsy-proven metastases (2) Clinical findings consistent with metastatic disease that trigger further treatment or a change in management (e.g., positive PET results followed by initiation of ADT or a change of drugs).

Death of disease: (1) Death as a direct result of disease progression (e.g., retroperitoneal lymphadenopathy leading to bilateral urinary obstruction and kidney failure) (2) Death as a direct result of treatment for the disease (e.g., drug toxicity). If you have any doubts, please add a comment in the “comments” column.

Data collection (please read this important information about how to fill selected individual cells):

1- Case: Please do not include any identifying information: Our IRB protocol only allows us to collect de-identified data. Instead, please identify your cases with the name of your institution (e.g., Indiana University 1, Indiana University 2, etc.). You can keep patient identifiers in your worksheet, but please do not share them with us.

2- Age: Age at the time of prostatectomy.

3- Sampling: “Entirely submitted” vs. “Representative”.

4- Location of the dominant nodule: If possible, per quadrant (e.g., left posterior, right posterior); otherwise “posterior/posterolateral” vs. “anterior” vs. “diffuse” (diffuse = involvement of 3 or more quadrants).

5. Configuration of the dominant nodule: Well-circumscribed vs. diffuse. Well-circumscribed nodules are characterized by the presence of a discrete nodular lesion that can usually be identified macroscopically/grossly. Diffuse nodules demonstrate “blurry” borders microscopically and are not identified as a discrete lesion grossly (they can be described as diffuse areas of induration instead).

6. Largest dimension of the dominant nodule: In millimeters (mm). Measured grossly (if apparent) or on glass slides)

7- Prostatectomy Gleason score: Please include only the number corresponding to the score (e.g., 9 instead of 4+5=9/5+4=9).

8- Prostatectomy Grade Group: Please include only numeric values (e.g., “4” instead of “Grade Group 4”).

9- Large cribriform components: This includes both intraductal and/or invasive cribriform glands ≥0.25 in diameter as recently defined or measuring at least twice the size of adjacent benign glands (PMID: 35145197).

10- Comedonecrosis: “Present” vs. “Absent”.

11- Lymphovascular invasion: “Present” vs. “Absent”.

12. Perineural invasion: Present vs. absent.

13- Adjuvant treatment: Post-RP adjuvant treatment (this excludes additional treatment given in the context of PSA recurrences or metastases).

14- Type of adjuvant treatment: Please include only the general type of treatment received (e.g., “radiation”).

15- PSA recurrence: “Yes” vs. “No”

16- Date of PSA recurrence or last follow up: Date when the definition of PSA recurrence is met (e.g., date of the second post-RP PSA measurement ≥ 0.2 ng/mL). If there is no PSA recurrence, please indicate the date of last follow up so that we can suppress/censor data in the outcome analysis.

17- Time to PSA recurrence: This should auto-fill when the date of PSA recurrence or last follow up is entered (see 8 above). If this does not auto-complete, please fill this cell manually in months, considering that the length of follow-up should be entered for patients without recurrence (i.e., in patients with post-RP follow up available, time to PSA recurrence should never be 0, even if the event was not observed)

18- Metastasis: “Yes” vs. “No”

19- Date of metastasis dx or last follow up: Date when metastasis is confirmed (e.g., date of the biopsy or imaging study that confirmed the disease). If there is no metastatic disease, please indicate the date of last follow up so that we can suppress/censor data in the outcome analysis.

20- Time to metastasis: This should auto-fill when the date of metastasis or last follow up is entered (see 16 above). If this does not auto-complete, please fill this cell manually in months, considering that the length of follow-up should be entered for patients without metastasis (i.e., in patients with post-RP follow up available, time to metastasis should never be 0, even if the event was not observed).

21- Death of disease: “Yes” vs. “No”. If this is unknown/uncertain, please fill as “No”.

22- Date of death of disease or last follow up: If this is uncertain, please use the date of the last clinical note. If there is no death of disease, please indicate the date of last follow up so that we can suppress/censor data in the outcome analysis.

23- Time to death of disease: This should auto-fill when the date of death of disease or last follow up is entered (see 19 above). If this does not auto-complete, please fill this cell manually in months, considering that the length of follow-up should be entered for patients without death of disease (i.e., in patients with post-RP follow up available, time to death of disease should never be 0, even if the event was not observed)
